# CYP1A1 Ile462Val Polymorphism Contributes to Lung Cancer Susceptibility among Lung Squamous Carcinoma and Smokers: A Meta-Analysis

**DOI:** 10.1371/journal.pone.0043397

**Published:** 2012-08-28

**Authors:** Ya-Nan Ji, Qin Wang, Li-jun Suo

**Affiliations:** 1 Jiangsu Province Hospital of Traditional Chinese Medicine, Nanjing, China; 2 Department of Respiratory Medicine, No. 81 Hospital of PLA, Nanjing, China; 3 Department of Respiratory Medicine, The Affiliated Linzi District People’s Hospital of Binzhou Medical University, Zibo, China; University of Oxford, United Kingdom

## Abstract

Many studies have examined the association between the CYP1A1 Ile462Val gene polymorphisms and lung cancer risk in various populations, but their results have been inconsistent. To assess this relationship more precisely, a meta-analysis was performed. Ultimately, 43 case-control studies, comprising 19,228 subjects were included. A significantly elevated lung cancer risk was associated with 2 Ile462Val genotype variants (for Val/Val vs Ile/Ile: OR = 1.22, 95% CI = 1.08–1.40; for (Ile/Val +Val/Val) vs Ile/Ile: OR = 1.15, 95% CI = 1.07–1.23) in overall population. In the stratified analysis, a significant association was found in Asians, Caucasians and lung SCC, not lung AC and lung SCLC. Additionally, a significant association was found in smoker population and not found in non-smoker populations. This meta-analysis suggests that the Ile462Val polymorphisms of CYP1A1 correlate with increased lung cancer susceptibility in Asian and Caucasian populations and there is an interaction with smoking status, but these associations vary in different histological types of lung caner.

## Introduction

Lung cancer remains the most lethal cancer worldwide, despite improvements in diagnostic and therapeutic techniques [1]. Its incidence has been increasing in many parts of world, particularly in China, which has become a major public health challenge all the world [2]. The mechanism of lung carcinogenesis is not understood. Although cigarette smoking is the major cause of lung cancer, not all smokers develop lung cancer [3], which suggests that other causes such as genetic susceptibility might contribute to the variation in individual lung cancer risk [4], [5]. Many environmental carcinogens require metabolic activation by drug-metabolizing enzymes. In recent years, several common low-penetrance genes have been implicated as potential lung cancer susceptibility genes.

Cytochrome P450 1A1 (CYP1A1) metabolizes several suspected procarcinogens, particularly polycyclic aromatic hydrocarbons (PAHs), into highly reactive intermediates [6]. These compounds bind to DNA to form adducts, which, if unrepaired, can initiate or accelerate carcinogenesis. Although PAHs are ubiquitous in the environment, notable sources of exposure that cause the greatest concern include smoking, air pollution, diet, and certain occupations [7]. Two functionally important nonsynonymous polymorphisms have been described for the CYP1A1 gene, a base substitution at codon 462 in exon 7, resulting in substitution of isoleucine with valine (Ile462Val (exon 7)) (National Center for Biotechnology Information single nucleotide polymorphism(SNP) identifier rs1048943; adenine (A) to guanine (G) substitution at nucleotide 2455(2455A.G)) and a point mutation (thymine (T) to cytosine (C)) at the MspI site in the 3′-untranslated region (rs4646903;3801T.C) [8]. The Ile462Val (exon 7) restriction site polymorphism resulted in three genotypes: a predominant homozygous (Ile/Ile), the heterozygote (Ile/Val), and the rare homozygous (Val/Val).

An association between CYP1A1 polymorphisms and lung cancer was first reported by Kawajiri and co-workers in 1990 among an Asian study population [9], after which many studies analyzed the influence of CYP1A1 polymorphisms on lung cancer risk; no clear consensus, however, was reached. Moreover, 3 meta-analyses have reported conflicting results. Houlston RS [10] found no statistically significant association between the MspI polymorphism and lung cancer risk in 15 studies, in a meta-analysis performed by Le Marchand L et al. [11] included only 11 studies, the Ile462Val (exon 7) polymorphism did not correlate with lung cancer risk. Shi X [12], however, noted a greater risk of lung cancer for CYP1A1 MspI and exon 7 polymorphism carriers in a meta-analysis that included only Chinese population in 15 studies.

A single study might not be powered sufficiently to detect a small effect of the polymorphisms on lung cancer, particularly in relatively small sample sizes. Various types of study populations and study designs might also have contributed to these disparate findings. To clarify the effect of the CYP1A1 Ile462Val (exon 7) polymorphism on the risk for lung cancer, we performed an updated meta-analysis of all eligible case-control studies to date and conducted the subgroup analysis by stratification according to the ethnicity source, histological types of lung caner and smoking status of case.

**Figure 1:**
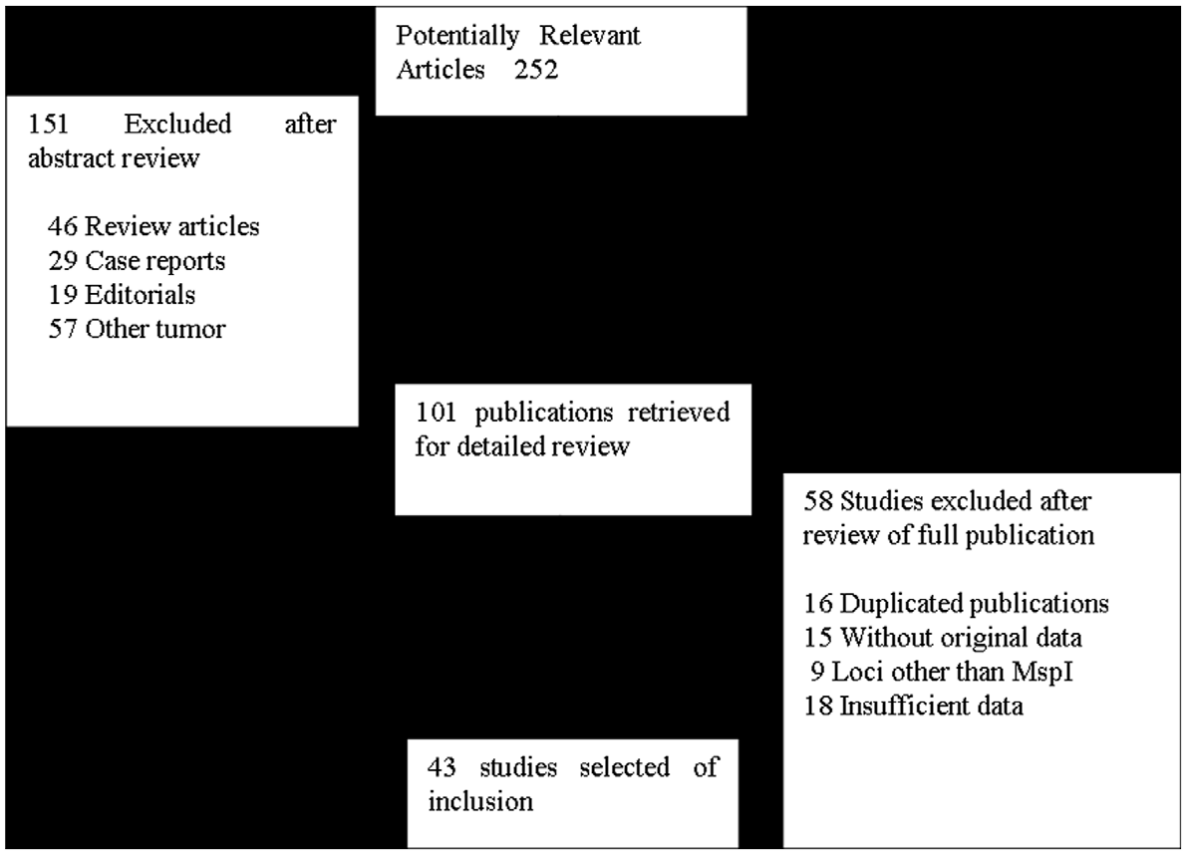
Flow diagram of the search strategy used.

## Materials and Methods

### 1. Publication Search

The electronic databases PubMed, Embase, Web of Science, and CNKI (China National Knowledge Infrastructure) were searched for studies to include in this meta-analysis, using the terms “CYP1A1,” “Cytochrome P450 1A1,” “polymorphism,” and “lung cancer.” An upper date limit of March 01, 2012 was applied; we used no lower date limit. The search was performed without any restrictions on language and was focused on studies that had been conducted in humans. We also reviewed the Cochrane Library for relevant articles. The reference lists of reviews and retrieved articles were hand searched simultaneously. When more than one of the same patient population was included in several publications, only the most recent or complete study was used in this meta-analysis.

**Table 1:** Distribution of CYP1A1 exon7 genotypes among lung cancer cases and controls included in this meta-analysis.

First author-year	Ethnicity(country of origin)	Total sample size (case/control)	Lung cancer cases			Controls		
			Ile/Val	Val/Val	Ile/Ile	Ile/Val	Val/Val	Ile/Ile
Nakachi K-1993	Asia(Japan)	31/127	11	6	14	44	4	79
Alexandrie AK-1994	Caucasian(Sweden)	296/329	16	0	280	23	0	306
Cantlay AM-1995	Caucasian(Edinburgh)	129/281	21	2	106	33	3	245
Kihara M-1995	Asia(Japan)	97/258	31	5	59	98	14	143
Ishibe N-1997	Mixed(Mexican andAfrican)	171/295	31	7	132	70	20	204
Hong YS-1998	Asia(Korean)	85/63	68	1	16	60	1	2
Taioli E-1998	Mixed populations	105/307	8	1	94	18	0	272
Sugimura H-1998	Asia(Japan)	247/185	94	28	125	84	7	94
Le Marchand L-1998	Mixed populations	341/456	68	6	263	105	13	335
Xue KX-1999	Asia(china)	103/131	31	18	54	36	11	36
Hu YL-1999	Asia(china)	59/132	33	7	19	102	9	21
London SJ-2000	Asia(China)	214/669	39	8	167	130	27	512
Song N-2001	Asia(China)	217/404	130	9	78	181	13	210
Ratnasinghe D-2001	Caucasian(USA)	282/324	36	3	243	48	3	273
Quinones L-2001	Caucasians(Chile)	60/174	35	10	15	52	14	54
Chen S-2001	Asia(china)	106/106	38	10	58	33	3	70
Xue KX-2001	Asia(china)	106/106	38	10	58	33	3	33
Zhou XW-2002	Asia(china)	92/98	66	11	15	65	6	65
Taioli E-2003	Mixed populations	110/707exon7	16	1	93	70	2	635
Dong CT-2004	Asia(china)	82/91	36	18	28	32	10	32
Yang XR-2004	Asia(China)	200/144	96	11	90	39	7	98
Sobti RC-2004	Asia(India)	100/76	67	29	4	53	15	8
Wenzlaff AS-2005	Caucasian(USA)	128/181	5#		124	14#		134
Wrensch MR-2005	Mixed populations	363/930exon7		64#	302		219#	711
Ng DP-2005	Asia(Singapore)	126/162	39	13	74	63	7	91
Larsen EJ-2005	Caucasians(Australia)	1050/581	84	8	958	27	2	552
Raimondi S-2005	Caucasians	175/723exon7		32#	143		67#	656
Raimondi S-2005-2	Asians	60/212 exon7		30#	30		96#	116
Li DR-2006	Asia(china)	150/152	104	14	32	105	8	105
Pisani P-2006	Asia(Thailand)	211/408	79	10	78	129	23	135
Yang MH-2007	Asia(Korea)	314/349	116	16	182	111	18	220
Cote ML-2007	Mixed populations	354/440	19	0	326	34	6	400
Yoon KA-2008	Asia(Korea)	213/213	76	10	127	87	10	116
Gallegos-Arreola-2008	Mixed populations	222/248	91	40	91	104	11	133
Shah PP-2008	Asia(India)	200/200		67#	133		44#	156
Kumar M-2009	Asia(India)	93/253	17	3	73	40	3	210
Cote ML-2009	Mixed populations	502/523	38	0	464	32	2	489
Klinchid J-2009	Asia(Thailand)	85/82		47#	33		42#	38
Timofeeva MN-2009	Caucasians (German)	619/1264	248	61	260	545	117	585
Wright CM-2010	Caucasians (Australian)	1040/784	103	8	929	40	3	741
Mota P-2010	Caucasian(Portugal)	175/217	38#		137	49#		168
Wang Z-2011	Asia(China)	72/90	9	26	37	25	11	54
Bai TY-2011	Asia(China)	106/250	66	15	25	172	24	54

&num; the number of the combined Ile/Val and Val/Val genotypes.

### 2. Inclusion Criteria

For inclusion, the studies must have met the following criteria: they (1) evaluated CYP1A1 Ile462Val (exon 7) gene polymorphisms and lung cancer risk; (2) were case-control studies or nested-case control study; (3) supplied the number of individual genotypes for the CYP1A1 Ile462Val (exon 7) polymorphisms in lung cancer cases and controls, respectively; and (4) demonstrated that the distribution of genotypes among controls were in Hardy-Weinberg equilibrium.

### 3. Data Extraction

Information was carefully extracted from all eligible publications independently by two authors according to the inclusion criteria. Disagreements were resolved through a discussion between the two authors. The following data were collected from each study: first author’s surname, year of publication, ethnicity, total numbers of cases and controls, and numbers of cases and controls who harbored the Ile462Val (exon 7) genotypes, respectively. We did not contact the author of the primary study to request the information. Ethnicities were categorized as Asian, Caucasian, and mixed. Histological type of lung cancer was divided to lung squamous carcinoma (SCC), adenocarcinoma (AC) and small cell lung cancer (SCLC) in our meta-analysis. The definition of smoking history is very complicated. The smoking histories covered different periods if changes in the number of cigarettes smoked per day or type of tobacco products occurred. According to the general standards, non-smokers were defined as subjects who had smoked less than 100 cigarettes in their lifetime. Although the precise definition of never-smoking status varied slightly among the studies, the smoking status was classified as non-smokers (or never smoker) and smokers (regardless of the extent of smoking) in our meta-analysis. We did not define any minimum number of patients to include a study in our meta-analysis.

**Figure 2:**
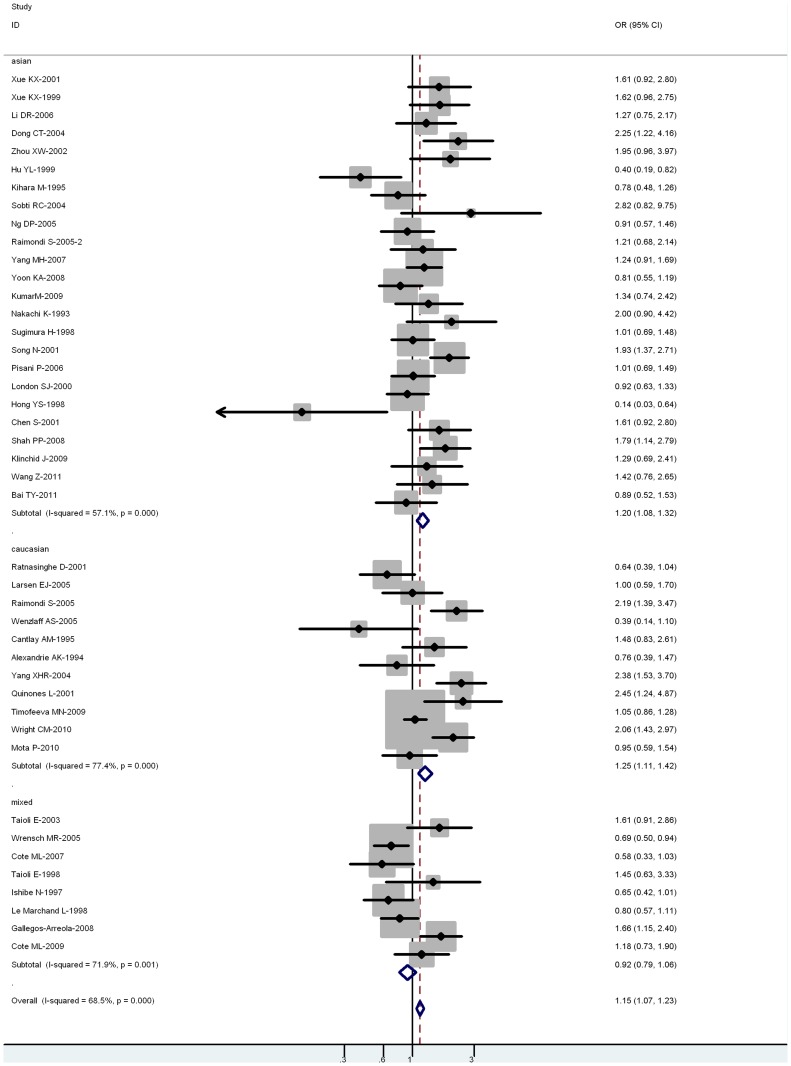
Forest plot (random-effects model) of lung cancer risk associated with CYP1A1 exon7 genotype for the combined Ile/Val and Val/Val vs Ile/Ile. Each box represents the OR point estimate, and its area is proportional to the weight of the study. The diamond (and broken line) represents the overall summary estimate, with CI represented by its width. The unbroken vertical line is set at the null value (OR = 1.0).

### 4. Statistical Analysis

OR (odds ratios) with 95% CIs were used to determine the strength of association between the CYP1A1 Ile462Val (exon 7) polymorphisms and lung cancer risk. We evaluated this risk with regard to combinations of variants (Ile/Val and Val/Val) versus the wild-type homozygotes (Ile/Ile).

The pooled ORs for the risk were calculated. Subgroup analyses were performed by ethnicity. Heterogeneity assumptions were assessed by chi-square-based Q-test [13]. A *P* value greater than 0.10 for the Q-test indicated a lack of heterogeneity among the studies. Thus, the pooled OR estimate of each study was calculated using the fixed-effects model (the Mantel–Haenszel method) [14]; otherwise, the random-effects model (the DerSimonian and Laird method) was used [15]. In addition, subgroup analysis stratified by ethnicity, gender and histological types of lung caner was also performed.

**Table 2:** Summary ORs for various contrasts of CYP1A1 exon7 gene polymorphisms in this meta-analysis.

Subgroup analysis	exon7 genotype				
	Contrast	studies	OR (95%) P_h_		
**Total**	Val/Val vs Ile/Ile(Ile/Val +Val/Val) vs Ile/Ile	43	1.22(1.08–1.40) 0.0041.15(1.07–1.23) 0.000		
**Ethnicity**					
Asian	Val/Val vs Ile/Ile(Ile/Val +Val/Val)vs Ile/Ile	24		1.22(1.16–1.59) 0.0161.20(1.09–1.33) 0.000	
Caucasian	Val/Val vs Ile/Ile(Ile/Val +Val/Val) vs Ile/Ile	11		1.24(1.17–1.43) 0.0901.25(1.11–1.42) 0.000	
Mixed population	Val/Val vs Ile/Ile(Ile/Val +Val/Val) vs Ile/Ile	8		0.84(0.77–1.03) 0.0900.92(0.79–1.06) 0.001	
**Histological type**					
SCC	Val/Val vs Ile/Ile(Ile/Val +Val/Val) vs Ile/Ile	12		1.38(1.12–1.66) 0.0041.42(1.18–1.70) 0.007	
AC	Val/Val vs Ile/Ile(Ile/Val +Val/Val) vs Ile/Ile	11		0.90(0.72–1.08) 0.0050.96(0.81–1.15) 0.003	
SCLC	Val/Val vs Ile/Ile(Ile/Val +Val/Val) vs Ile/Ile	7		0.84(0.68–1.08)0.0680.78(0.53–1.14) 0.039	
**Smoking status**					
Smoking	Val/Val vs Ile/Ile(Ile/Val +Val/Val) vs Ile/Ile		1.60(1.20–2.09) 0.0061.62(1.24–2.11) 0.004		
Non-smoking	Val/Val vs Ile/Ile(Ile/Val +Val/Val) vs Ile/Ile		1.02(0.84–1.39) 0.0091.07(0.88–1.31) 0.002		

P_h_ P value of Q-test for heterogeneity test.

One-way sensitivity analyses were performed to determine the stability of the results–each individual study in the meta-analysis was omitted to reflect the influence of the individual dataset on the pooled OR [16].

Potential publication biases were estimated by funnel plot, in which the standard error of log (OR) of each study was plotted against its log (OR). An asymmetrical plot suggests a publication bias. Funnel plot asymmetry was assessed by Egger’s linear regression test, a linear regression approach that measures the funnel plot asymmetry on a natural logarithm scale of the OR. The significance of the intercept was determined by t-test, as suggested by Egger (P<0.05 was considered a statistically significant publication bias) [17].

All calculations were performed using STATA, version 10.0 (Stata Corporation, College Station, TX).

**Figure 3:**
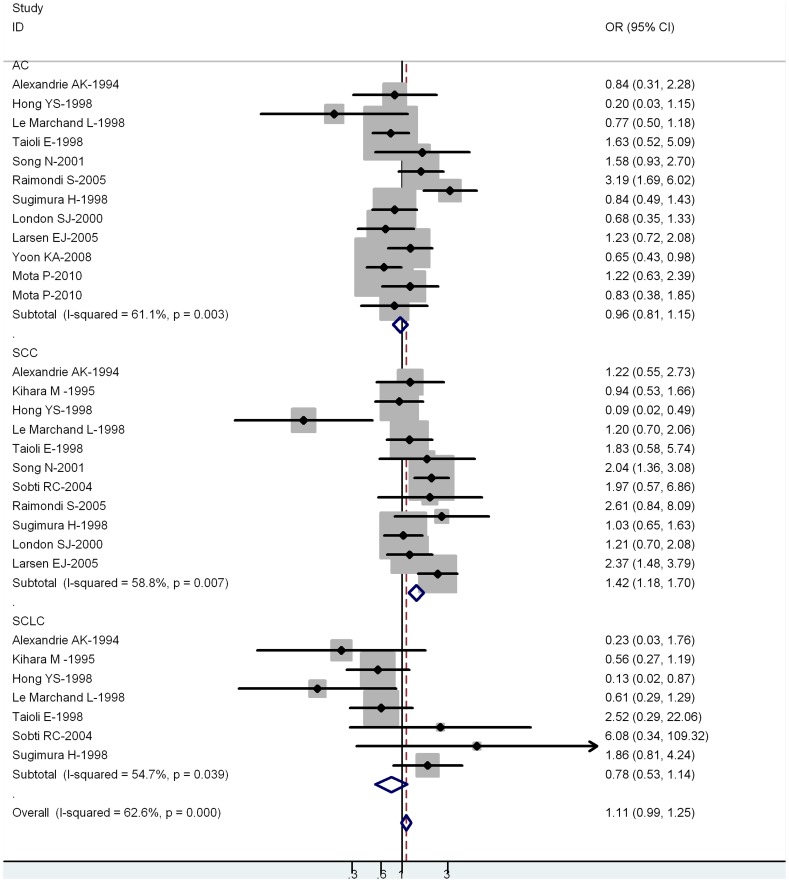
Forest plot (random-effects model) of lung cancer risk associated with CYP1A1 exon7 genotype for the combined Ile/Val and Val/Val vs Ile/Ile by histological types of lung cancer.

**Table 3:** Distribution of CYP1A1 exon7 genotypes among cases and controls stratified by histological types of lung cancer.

First author-year	Ethnicity(country of origin)	Histology (Scc/Ac/Sclc)	Lung cancer cases			Controls		
			Ile/Val	Val/Val	Ile/Ile	Ile/Val	Val/Val	Ile/Ile
Alexandrie AK-1994	Caucasian(Sweden)	SCC	9	0	98	23	0	306
		AC	5	0	79	23	0	306
		SCLC	1	0	57	23	0	306
Kihara M -1995	Asia(Japan)	SCC	23	2	34	98	14	143
		SCLC	8	3	25	98	14	143
Hong YS-1998	Asia(Korean)	SCC	19	1	7	60	1	2
		AC	24	0	4	60	1	2
		SCLC	12	0	3	60	1	2
Le Marchand L-1998	Mixed populations	SCC	21	1	52	105	13	335
		AC	31	3	126	105	13	335
		SCLC	8	1	42	105	13	335
Sugimura H-1998	Asia(Japan)	SCC	46	15	61	84	7	94
		AC	27	8	43	84	7	94
		SCLC	13	5	10	84	7	94
Taioli E-1998	Mixed populations	SCC	3	1	33	18	0	272
		AC	3	1	37	18	0	272
		SCLC	1	0	6	18	0	272
London SJ-2000	Asia(China)	SCC	18	2	54	130	27	512
		AC	11	0	53	130	27	512
Song N-2001	Asia(China)	SCC	81	4	45	181	13	210
		AC	35	3	26	181	13	210
Sobti RC-2004	Asia(India)	SCC	50	17	4	53	15	8
		SCLC	12	12	0	53	15	8
Larsen EJ-2005	Caucasians(Australia)	SCC		53#	426	27	2	552
		AC		29#	450	27	2	552
Raimondi S-2005	Caucasians	SCC		4#	15		67#	656
		AC		15#	46		67#	656
Yoon KA-2008	Asia(Korea)	AC	54	7	112	87	10	116
Mota P-2010	Caucasian(Portugal)	AC		15#	42		49#	168
		SCC		9#	37		49#	168

&num; the number of the combined Ile/Val and Val/Val genotypes.

## Results

### 1. Study Characteristics

Two hundred and fifty-two potentially relevant citations were reviewed, and 43 publications met the inclusion criteria and included in our meta-analysis [18]–[59]. The study search process is shown in Figure 1. Table 1 presents the principal characteristics of these studies. Raimondi’s study [43] sorted the data for Caucasians and Asians; therefore, each group in the study was considered separately in the pooled subgroup analyses.

**Figure 4:**
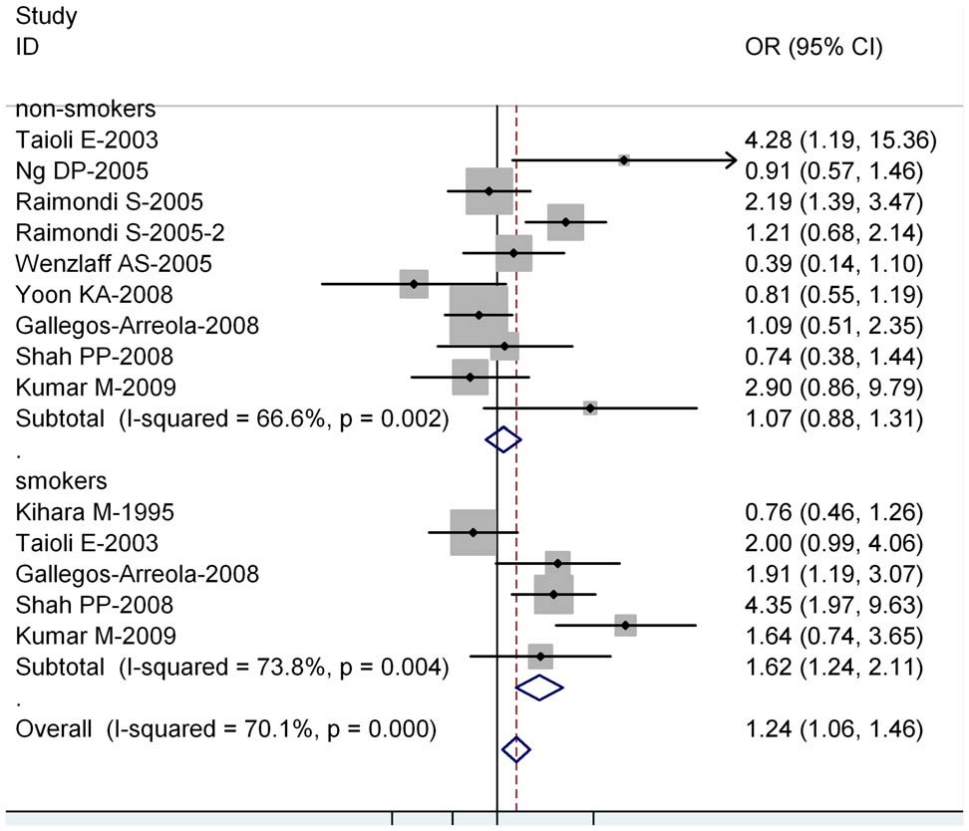
Forest plot (random-effects model) of lung cancer risk associated with CYP1A1 exon7 genotype for the combined Ile/Val and Val/Val vs Ile/Ile stratified by smoking status of population.

**Table 4:** Distribution of CYP1A1 exon7 genotypes among cases and controls stratified by smoking status.

First author-year	Ethnicity(country of origin)	Smoking status	Lung cancer cases			Controls		
			Ile/Val	Val/Val	Ile/Ile	Ile/Val	Val/Val	Ile/Ile
Kihara M-1995	Asia(Japan)	Smokers	31	5	59	70	11	101
Taioli E-2003	Mixed populations	Non-smokers	4	0	7	35	0	262
		Smokers	12	1	77	26	1	320
Ng DP-2005	Asia(Singapore)	Non-smokers	39	13	74	63	7	91
Raimondi S-2005	Caucasians	Non-smokers		32#	143		67#	656
Raimondi S-2005-2	Asians	Non-smokers		30#	30		96#	116
Wenzlaff AS-2005	Caucasian(USA)	Non-smokers		5#	124		14#	134
Yoon KA-2008	Asia(Korea)	Non-smokers	76	10	127	87	10	116
Gallegos-Arreola-2008	Mixed populations	Non-smokers	8	8	16	55	11	72
		Smokers	83	32	75	49	0	61
Shah PP-2008	Asia(India)	Non-smokers		16#	64		35#	103
		Smokers		51#	69		9#	53
Kumar M-2009	Asia(India)	Non-smokers	4	1	7	28	2	122
		Smokers	14	2	66	12	1	88

&num; the number of the combined Ile/Val and Val/Val genotypes.

Of the 43 publications, 35 were published in English and 8 were written in Chinese. The sample sizes ranged from 104 to 1824. All cases were histologically confirmed. The controls were primarily healthy populations and matched for age, ethnicity, and smoking status, 15 studies were hospital-based control and 28 were population-based control. There were 24 groups of Asians, 11 groups of Caucasians, and 8 mixed populations.

### 2. Meta-analysis Results

For all studies in the meta-analysis, the genotype, an increased risk for lung cancer was associated with 2 Ile462Val variants (for Val/Val vs Ile/Ile: OR = 1.22, 95% CI = 1.08–1.40, *P = *0.004 for heterogeneity; for Ile/Val and Val/Val combined vs Ile/Ile: OR = 1.15, 95% CI = 1.07–1.23, *P*<0.001 for heterogeneity) (Figure 2).

**Figure 5:**
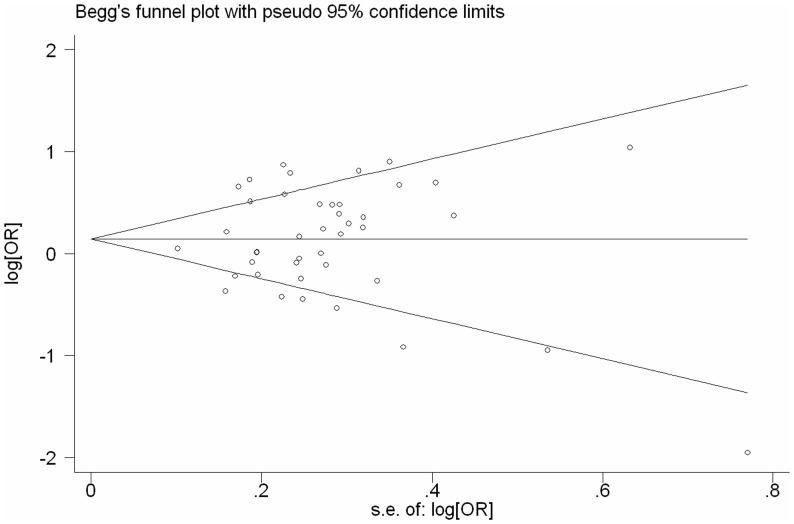
Begg’s funnel plot of CYP1A1exon7 gene polymorphism and lung cancer risk for the combined Ile/Val and Val/Val vs Ile/Ile.

In the stratified analysis by ethnicity, the risk was higher in Asian carriers of Val/Val vs Ile/Ile (OR = 1.22, 95% CI = 1.16–1.59; *P = *0.016 for heterogeneity) and Ile/Val and Val/Val combined vs Ile/Ile (OR = 1.20, 95% CI = 1.09–1.33; *P*<0.001 for heterogeneity). A significant association was also observed in Caucasian carriers of Val/Val vs Ile/Ile (OR = 1.24; 95% CI = 1.17–1.43; *P = *0.090 for heterogeneity) and Ile/Val and Val/Val combined vs Ile/Ile (OR = 1.25; 95% CI = 1.11–1.42; *P*<0.001 for heterogeneity). However, no significant associations were observed in mixed populations for both Val/Val vs Ile/Ile (OR = 0.84; 95% CI = 0.77–1.03; *P = *0.090 for heterogeneity) or Ile/Val and Val/Val combined vs Ile/Ile (OR = 0.92; 95% CI = 0.79–1.06; *P = *0.001 for heterogeneity) (Table 2).

Twelve-one out of 43 studies examined the association of CYP1A1 exon 7 genotype and the risk of different histological types of lung cancer including SCC, AC and SCLC (Table 3). Among lung SCC, significantly increased risks were observed for both Val/Val vs Ile/Ile (OR = 1.38; 95% CI = 1.12–1.66; *P = *0.004 for heterogeneity) or Ile/Val and Val/Val combined vs Ile/Ile (OR = 1.42; 95% CI = 1.18–1.70; *P = *0.007 for heterogeneity. However, among lung AC and SCLC, no significant associations were observed for both Val/Val vs Ile/Ile or Ile/Val and Val/Val combined vs Ile/Ile (Figure 3).

Ten out of 40 studies included the association of CYP1A1 exon 7 genotype and lung caner risk stratified by smoking status (non-smokers or never smokers and smokers) (Table 4). For smokers, significantly increased risks were observed for both Val/Val vs Ile/Ile (OR = 1.60; 95% CI = 1.20–2.09; *P = *0.006 for heterogeneity) and Ile/Val and Val/Val combined vs Ile/Ile (OR = 1.62; 95% CI = 1.24–2.11; *P = *0.004 for heterogeneity). However, for non-smokers, no significant associations were observed for both Val/Val vs Ile/Ile (OR = 1.02; 95% CI = 0.84–1.39; *P = *0.009 for heterogeneity) or Ile/Val and Val/Val combined vs Ile/Ile (OR = 1.07; 95% CI = 0.88–1.31; *P = *0.002 for heterogeneity) (Figure 4).

### 3. Sensitivity Analyses

A single study involved in the meta-analysis was deleted each time to reﬂect the inﬂuence of the individual data-set to the pooled ORs, and the corresponding pooled ORs were not materially altered (data not shown).

### 4. Publication Bias

Begg’s funnel plot and Egger’s test were performed to identify any publication bias. The funnel plots did not exhibit any patent asymmetry (Figure 5). By Egger’s test–used to provide statistical evidence of funnel plot symmetry–there was no evidence of publication bias (P = 0.733 for publication bias).

## Discussion

CYP genes are large families of endoplasmic and cytosolic enzymes that catalyze the activation and detoxification, respectively, of reactive electrophilic compounds, including many environmental carcinogens (e.g., benzo[a] pyrene). CYP1A1 is a phase I enzyme that regulates the metabolic activation of major classes of tobacco procarcinogens, such as aromatic amines and PAHs [6]. Thus, it might affect the metabolism of environmental carcinogens and alter the susceptibility to lung cancer. This meta-analysis explored the association between the CYP1A1 exon7 gene polymorphisms and lung cancer risk, and performed the subgroup analysis stratified by ethnicity, histological types of lung caner, gender and smoking status of case and control population. Our results indicated a significant association between CYP1A1 exon7 gene polymorphism and lung cancer risk Asians, Caucasians, lung SCC and Female population, no significant association was found in mixed population, lung AD, lung SCLC and Male population. Additionally, a significant association was found in smoker population and not in non-smoker populations.

When stratified according to ethnicity, a significantly increased risks were identified among Asians and Caucasians for the 2 exon 7 genotype variants, however no significant association was found in mixed population. These findings indicate that polymorphisms of CYP1A1 exon 7 polymorphism may be important in specific ethnicity of lung cancer patients. Population stratification is an area of concern, and can lead to spurious evidence for the association between the marker and disease, suggesting a possible role of ethnic differences in genetic backgrounds and the environment they lived in [60]. In fact, the distribution of the less common Val allele of exon 7 genotype varies extensively between different races, with a prevalence of ∼25% among East Asians, ∼5% among Caucasians and ∼15% among other population. In addition, in our meta-analysis the between-study heterogeneity was existed in overall population, the subgroup of Asian and Caucasian for exon 7 genotypes. The I-squared value of Asian group is 57%, which is lower than the I-squared values for the Caucasians and mixed population studies, suggesting less heterogeneity among the Asian populations. Therefore, additional studies are warranted to further validate ethnic difference in the effect of this functional polymorphism on lung cancer risk.

There are growing biological and epidemiological data to suggest that different lung cancer pathological subtypes, particularly the two most common, are distinct etiological entities that should be analyzed separately [61]. When subgroup analyses by pathological types were considered, CYPIAl exon7 variant alleles were found to be associated with a 1.4 fold increase in the risk of lung SCC. However, for lung AC and SCLC, no significant association was found. Our findings were consistent with the Le Marchand L et al study [26] with largest sample sizes of case and control. Le Marchand et al. hypothesized that genetic susceptibility to PAHs predominantly caused lung SCC and nitrosamines caused lung AC. With introduction of filter-tipped cigarettes, probably decreased smokers’ exposure to PAHs and increased their exposure to nitrosamines, decreasing trend of SCC, relative to the increase in AC indirectly supports this hypothesis [62]. Different carcinogenic processes may be involved in the genesis of various tumor types because of the presence of functionally different CYP1Al exon7 gene polymorphisms. However, the possible molecular mechanisms to explain these histology-specific differences in the risk of lung cancer remain unresolved.

As we know, aside from genetic factor, smoking is the major risk factor of lung cancer. Most studies out of 40 studies reported information on smoking habits of cases and controls, however only ten eligible publications provided non-smokers information. Our meta-analysis results showed that a significantly increased risk was found to be associated with the CYP1A1 exon 7 gene polymorphisms and lung cancer risk in smokers, however, no significant association was found among non-smokers. The I-squared value of non-smokers groups is lower than the I-squared values for the smoker population studies, suggesting less heterogeneity among non-smokers populations. Tobacco smoke contains many of carcinogens and procarcinogens, such as benzopyrene and nitrosamine. These compounds are metabolized by the phase I enzymes including CYP family enzymes and converted to inactivemetabolites by the phase II enzymes. Our results should indicate the interaction between CYP1A1 exon 7 gene polymorphisms and smoking in the development of lung carcinoma. However, the association between the extent of smoke exposure and lung caner risk was not clear, further studies with larger sample size are needed to provide insights into the association.

Some limitations of this meta-analysis should be acknowledged. First, heterogeneity can interfere with the interpretation of the results of a meta-analysis. Although we minimized this likelihood by performing a careful search of published studies, using explicit criteria for a study’s inclusion and performing strict data extraction and analysis, significant interstudy heterogeneity nevertheless existed in nearly every comparison. The presence of heterogeneity can result from differences in the selection of controls, age distribution, and prevalence of lifestyle factors. Further, only published studies were included in this meta-analysis. The presence of publication bias indicates that non-significant or negative findings might be unpublished. Finally, in the subgroup analyses, different ethnicities were confused with other population, which may bring in some heterogeneity. As studies among the Indians and Africans are currently limited, further studies including a wider spectrum of subjects should be carried to investigate the role of these variants in different populations.

In conclusion, the results of our meta-analysis have provided the comprehensive and convincing evidence that CYP1A1 exon 7 polymorphisms are an important modifying factor in determining susceptibility to lung cancer. The effect of CYP1A1 exon 7 gene polymorphisms is diverse by the subgroup analysis stratified by ethnicity, histological types of lung caner and gender of case and control population. More importantly, our study confirms that there is an interaction between two genotypes of CYP1A1 exon 7 gene polymorphisms and smoking. For future studies, strict selection of patients, well-matched controls and larger sample size will be required. Moreover, gene–gene and gene–environment interactions should also be considered.
